# Migraine Aura: Retracting Particle-Like Waves in Weakly Susceptible Cortex

**DOI:** 10.1371/journal.pone.0005007

**Published:** 2009-04-01

**Authors:** Markus A. Dahlem, Nouchine Hadjikhani

**Affiliations:** 1 Institut für Theoretische Physik, Technische Universität Berlin, Berlin, Germany; 2 Klinik für Neurologie II, Otto-von-Guericke-Universität Magdeburg, Magdeburg, Germany; 3 Leibniz Institute für Neurobiologie, Magdeburg, Germany; 4 Martinos Center for Biomedical Imaging, Massachusetts General Hospital, Harvard Medical School, Charlestown, Massachusetts, United States of America; 5 Brain Mind Institute, EPFL, Lausanne, Switzerland; Tel Aviv University, Israel

## Abstract

Cortical spreading depression (SD) has been suggested to underlie migraine aura. Despite a precise match in speed, the spatio-temporal patterns of SD observed in animal cortex and aura symptoms mapped to the cortical surface ordinarily differ in aspects of size and shape. We show that this mismatch is reconciled by utilizing that both pattern types bifurcate from an instability point of generic reaction-diffusion models. To classify these spatio-temporal pattern we suggest a susceptibility scale having the value σ = 1 at the instability point. We predict that human cortex is only weakly susceptible to SD (σ<1), and support this prediction by directly matching visual aura symptoms with anatomical landmarks using fMRI retinotopic mapping. Moreover, we use retinal SD to give a proof of concept of the existence of this instability point and describe how cortical susceptibility to SD must be adjusted for migraine drug testing. Close to the instability point at σ = 1 the dynamical repertoire of cortical tissue is increased. As a consequence, the picture of an engulfing SD that became paradigmatic for migraine with aura needs to be modified in most cases towards a more spatially confined pattern that remains within the originating major gyrus or sulcus. Furthermore, we discuss the resulting implications on migraine pharmacology that is hitherto tested in the regime (σ>1), and potentially silent aura occurring below a second bifurcation point at σ = 0 on the susceptible scale.

## Introduction

**Figure 1:**
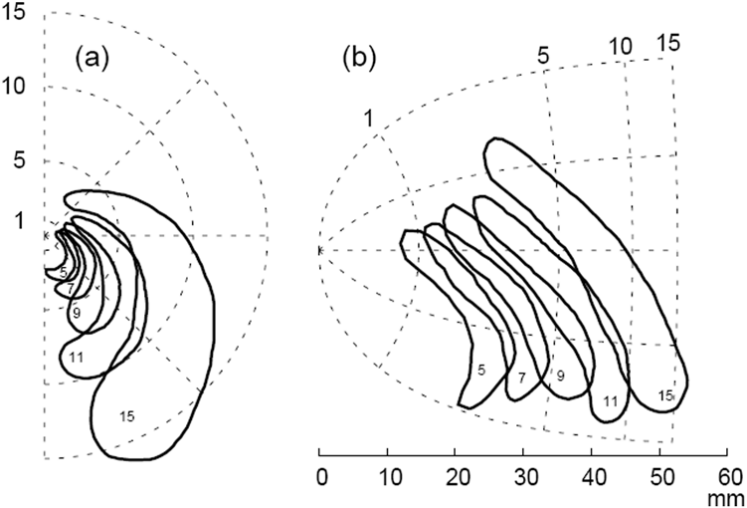
Typical propagation pattern of a visual migraine aura. (a) Right visual hemifield (dotted polar grid) with five subsequent sketched “snapshots” of a traveling visual migraine aura symptom in the shape of a crescent pattern. Numbers inside the scotom gives the time passed (in minutes) since first occurrence. Data is taken from Ref. [1] (b) Visual field disturbance shown by reversed retinotopic mapping projecting the affected area onto a flat model of the primary visual cortex.1.

Migraine aura is a collection of transient neurological symptoms characterized by a gradual onset as the distinctive clinical feature. It may be classified into sensory and cognitive modalities. Visual aura predominate, usually consisting of a distortion in the visual field often characterized by an expanding zigzag pattern at the leading front and a scotoma in the back [1], [2], [3], [4], [5], [6] (Fig. 1 (a)). Direct correlations between aura percepts and neural properties have been demonstrated, e. g., the typical zigzag patterns are reflected in reversed cortical feature maps [2], [7], [8]. While the pseudohallucinatory percept during the aura (visual or other) is specific of the affected sensory modality and is independent of etiology [9], the spatio-temporal course of aura progression is a clear signature of the underlying pathological process.

Reverse retinotopic mapping of aura symptoms reveals a constant propagation speed of about 3 mm/min on the cortical surface [1], [6] (Fig. 1 (b)). The remarkable slow velocity fits with the pace of spreading depression (SD), a profound but transient all-or-none type process characterized by redistribution of ions across cell membranes and nearly complete neuronal depolarization [10], [11]. This suggests that both phenomena rely on the same propagation mechanism [12]. Evidence for further links between SD and migraine are complex and mostly indirect [13], and whether the migraine headache is initiated by SD remains an active debate.

Despite the precise match in speed of mapped aura symptoms and SD, both processes ordinarily differ in aspects of size and shape on the cortical surface. While SD waves usually invade the entire gray matter region and stop only at the border with white matter—at least if observed in the most prone brain regions, the hippocampus and neocortex of nonprimate mammals—migraine aura symptoms, in contrast, seem to be more spatially confined. This can be deduced from the fact that visual symptoms often last not longer than 20 min corresponding to a propagation distance of 60 mm, the length of the early visual areas located along the calcarine sulcus (Fig. 1). It is the central result of this article to show that this mismatch in size and shape between mapped aura symptoms and SD propagation may be reconciled by utilizing that both pattern types occur in a generic reaction-diffusion model but are separated by a bifurcation, that is, a sudden qualitative change in the spatio-temporal SD pattern after only a small smooth change made to cortical susceptibility to SD. Our predictions are supported by directly matching visual aura symptoms with anatomical landmarks using fMRI retinotopic mapping.

Our results lead us to the conclusion that SD in humans is much closer to a bifurcating instability point of pattern formation than in nonprimate mammals. From a synergetics point of view, the brain is in general viewed as a self-organizing pattern forming system that operates close to instability points [14]. In the case of migraine aura, the crucial instability point separates transient from sustained wave propagation. Being close to the instability point dramatically changes the dynamical repertoire. This factor, as will be discussed, should have important implications on the design of migraine drug tests. Moreover, it may favor the hypothesis of the occurrence of silent aura in diagnosed forms of *migraine without aura* [15].

## Results

The visual aura symptoms typically affect only a part, albeit large part, of a visual hemifield. The affected area forms an expanding circular arc often centred close to the fovea. In Fig. 1 (a), a sequence of subsequent migraine aura “snapshots” visualizes the typical course of a visual disturbance in the right visual hemifield. The perimetric data is taken from Lashley [1] and the corresponding spatio-temporal pattern in the primary visual cortex (V1) is obtained by reversed retinotopic mapping (Fig. 1 (b)). The crescent pattern in the visual hemifield translates into a wave segment resembling a “particle-like wave” [16], as described in the next subsection. From this pattern, we can estimate an average length of the wave front of about 35 mm and a propagation speed of 3 mm/min. Therefore, such a wave segment temporarily recruits a total of about 2100 mm^2^ cortical surface within 20 min into the depolarized SD state, that is, only approximately 1.7% of the surface of one human cortical hemisphere.

### Susceptibility scale based on wave instabilities

**Figure 2:**
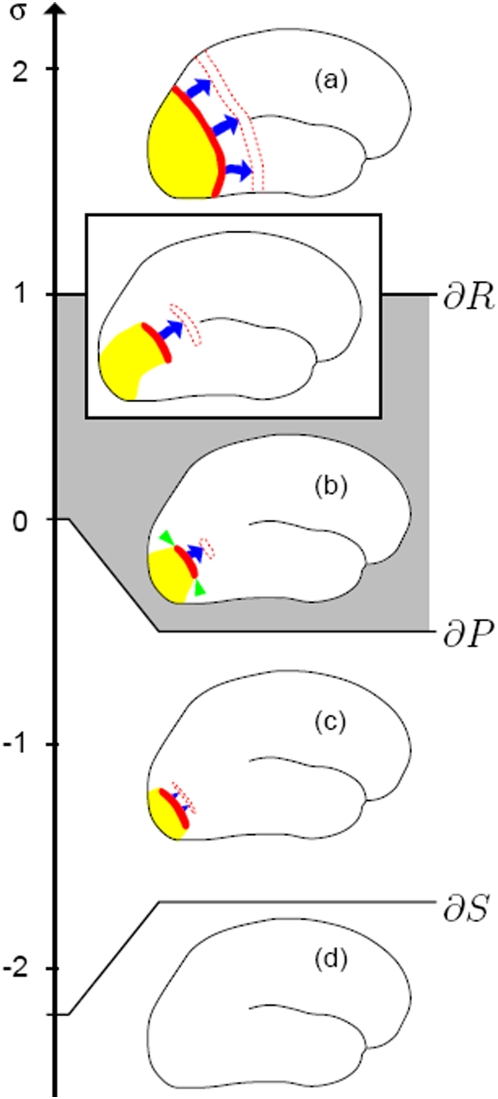
Schematic view of the spatio-temporal course of a reaction-diffusion wave for different tissue susceptibility values σ: wave front (red), recovery phase (yellow), blue arrows indicate normal velocity, future location is dashed (red). (a) sustained wave, (b) retracting wave, indicated by green arrow heads, (c) collapsing wave, (d) no spread. The gray σ interval is defined as weakly susceptible.

Transient and spatially confined waves were first suggested to cause aura symptoms in a descriptive mathematical model considering the motion of curves with free ends [17]. These curves represent segments of excitation fronts with two open ends, as shown in Fig. 1 (b). Furthermore, unstable—and thus also transient and spatially confined—waves, termed *particle-like waves* have been found and studied in the chemical Belousov-Zhabotinskii (BZ) reaction and their spatio-temporal dynamics are described by reaction-diffusion equations [16], [18], [19]. Particle-like wave propagation differs significantly from the current view of SD as a pattern engulfing posterior cortex (Fig. 2 (a)).

We suggest to introduce a macroscopic susceptibility scale σ to classify such spatio-temporal reaction-diffusion patterns in excitable media that are weakly susceptible to SD wave propagation. A two-point definition is used for calibrating this scale, whereby σ = 1 represents particle-like waves and σ = 0 the propagation boundary (see Methods). These two points are defined each by a bifurcation. The value σ = 1 separates excitable media with capacity to propagate growing waves segments (σ>1) from those where only retracting waves segments (σ<1) occur. When susceptibility changes to a value below σ = 0, the amplitude of the wave decreases so that a wave segment not only retracts from its sides (decreasing length as indicated by green arrow heads in Fig. 2 (b)) but also its wave profile collapses (decreasing width).

The two bifurcations at σ = 0 and σ = 1 are generic in the sense that they apply to excitable media based on reaction-diffusion mechanisms irrespective of the particular model. In Fig. 2, generic spatio-temporal reaction-diffusion patterns are classified into four intervals based on a linear scale between the points σ = 0 and σ = 1. The linear scale and the locus of further bifurcation points on this scale depend on the specific reaction-diffusion model. We used the FitzHugh-Nagumo system and fixed all parameters but the threshold β such that the experimentally observed re-entrant pattern of retinal SD, which performs a complex meandering [20], is obtained at σ>2 (Methods) [21], [22].

Four susceptibility intervals are relevant for reconciling the mismatch in size and shape between mapped aura symptoms and SD propagation. They are ordered by decreasing susceptibility: (a) (σ>1): sustained waves, (b) (1>σ>0): retracting waves, (c) (σ<0): collapsing waves, (d) (σ<−2.2): no spread.

The regime (a) has the highest susceptibility to spreading phenomena due to the lowest threshold values among the four intervals. The spatio-temporal patterns obtained in (a) show the typical course of SD waves observed in animal experiments. In particular, an SD wave, initiated at the occipital pole and propagating in anterior direction, will eventually engulf the whole cortical surface. In this regime, wave segments with free open ends curl in to form rotors (spiral shaped waves), therefore the lower bound σ = 1 is called the rotor boundary (∂*R*) [21]. The rotor boundary is marked by the occurrence of particle-like waves. Adhering strictly to the definition of particle-like waves as a wave form with natural length and shape that will either grow or decay when perturbed, the location of the rotor boundary in parameter space is wave size dependent [18]. In the limit of large wave segments (critical fingers [23], [24]) susceptibility approaches a lower bound that is used as the defining point for calibrating σ.

In the intervals (b) and (c) transient waves forms occur. In (b), the interval with higher susceptibility among (b) and (c), 2D wave segments with free open ends, such as shown in Fig. 1 (b), eventually disappear because open ends retract and thereby constantly reduce the instantaneous size of excitation. The wave segments in Fig. 1 (b) grows by about 30% in its length within 10 min. This indicates that σ>1. Comparing the duration of 10 min with the rotation period of 2.5 min of freely cycling SD waves (spiral waves observed at about σ = 2) [20] reveals that σ is close to one, or, in words, the growth is very slow. For larger values of σ, each open end of the SD wave segment curls in and performs four complete cycles thereby dramatically increasing the total area invaded by the wave. Cortical spiral SD waves have not been observed but reverberating cortical SD waves [25]. Reverberating waves are also spiral-shaped but their center is anchored to a lesion. Their rotation period depends on the size of the lesion. For small lesions the rotation period converges to the absolute refractory period, which is also considerably shorter than 10 min. In conclusion, 30% change of size within 10 min is rather negligibly small and therefore σ is close to one. If σ is close to one, the curvature of both cortex (see next section) and wave segment [17] can change the normal spatio-temporal development, for example, the wave segment can temporally grow (shrink) although σ<1 (σ>1). This can explain why the wave segment in Fig. 1 (b) eventually disappeared. That it did disappear can be concluded, because neurological symptoms where reported by Lashley to never last longer than 20 min.

In susceptibility interval (c) the reaction-diffusion equations describe rather a process of facilitated diffusion than travelling wave processes in excitable media. For this reason, the boundary between (b) and (c) is called propagation boundary (∂*P*) [21], [24]. In (c), the spatio-temporal development of an initial perturbation of cortical homeostasis, for example a massive localized increase in the extracellular potassium concentration, is similar to the development caused by mere passive diffusion. The main difference ist that the spatially elevated distribution collapses slower due to additional reactions sources and this process can be directed forming a transient wave [26]. In regime (d), an initially imposed spatially localized elevated distribution collapses without broadening (spreading boundary (∂*S*)) because the reaction part provides mainly a sink that decreases this elevated distribution faster than it is transported outwards by diffusion [26].

### Effects of gyrification on reaction-diffusion waves

Critical properties of reaction-diffusion waves such as retracting particle-like wave propagation in the weakly susceptibility domain 1>σ>0 are modulated by the bending of the cortical surface. This can be deduced from experimental and theoretical [27], [28], [29], [30] studies of the chemical BZ model systems of reaction-diffusion waves on curved surfaces in the regime of weakly excitable media. Weak excitability is not strictly defined but usually refers to values close to σ = 1. In these systems, it is shown that propagation depends crucially on the geometric properties of the surface. As a consequence, we can predict that a correlation must exist between anatomical landmarks and the course of aura symptoms if migraine aura is caused by a reaction-diffusion process.

In this subsection, we consider the gross gyral morphology in relation to the typical aura onset, course and ending. But before, we refer to a particular curvature-induced phenomenon that provides a mechanism how wave segments can emerge in the first place. It was shown that the wave front can undergo a critical deformation above which propagation is blocked [31]. A broken wave front is needed to distinguish spatio-temporal pattern obtained in the susceptibility intervals (σ>1) and (1>σ>0). The evolution of closed wave fronts does not differ much until the front breaks open, for instance due to a local curvature-induced excitation block. Then the resulting open ends will either grow or retract if the susceptibility σ is in the interval (σ>1) and (1>σ>0), respectively.

If migraine aura is caused by retracting reaction-diffusion waves (1>σ>0) that are guided by anatomical landmarks, the main course of the neurological symptoms within different people can be similar, because many studies of human cytoarchitecture show that sensory and motor areas have some relationship to the gross sulcal and gyral morphology. In some cases very precise correlations between sulci and functional entities could be demonstrated, most prominent is the calcarine sulcus as a landmark of the primary visual cortex (V1) [32]. Furthermore, the primary auditory cortex has a clear spatial relationship with Heschl's gyrus [33], [34], and the motor cortex can be identified by the position of the central sulcus [35]. Yet a substantial interindividual and interhemisphere variability in both size and location of anatomical landmarks is observed [36], and major sulci and gyri are individually composed of smaller gyral folds and sulci indents, which provides a variability for individual local characteristics of the spatio-temporal aura symptoms.

Due to calcarine sulcus' precise landmark identification of V1 [32], its geometric properties are best suited for comparison with visual aura symptoms. Furthermore, its retinotopic mapping of visual input is well studied in human [37], [38], [39], [40]. We therefore consider the gross morphology of the calcarine sulcus and the relative position of V1 in relation to the typical onset, course and ending of crescent shaped visual aura as shown in Fig. 1.

#### Onset

Most of the crescent shaped aura pattern start in one visual hemifield close to the fovea (center of gaze). The neural representation of the fovea is located at the occipital pole often extending about 10 mm onto the lateral convexity. The calcarine sulcus is formed by the cuneus and lingual gyrus on the medial surface and runs forward to the corpus callosum. Approximately two-thirds of V1 lies within the calcarine sulcus walls [32]. A difference of visual angle between the onset of aura symptoms and the fovea corresponds to a cortical distance of about 1 cm (see Fig. 1) because of the large linear cortical magnification factor *M* (see Methods) close to the fovea. Therefore, the crescent aura symptoms start near the entrance of the calcarine sulcus.

#### Course

Typical crescent pattern propagate along the horizontal hemimeridian towards the visual periphery. The pattern extends into both quadrants of the visual hemifield, which is a sign that it is arises in V1, V3A or V8, which are the only visual areas where the two quadrants of the visual hemifield are not split along the horizontal hemimeridian [41]. Other extrastriate visual cortical areas represent the two quadrants of a visual hemifield in dorsal and ventral areas that are connected only close to the fovea. The pattern is caused most probably in early visual areas because orientation selective cells with moderate receptive field sizes are only found there. They represent the individual edges of the zigzag aura percept at the propagating front [8].

The locus of the neural representation of the horizontal hemimeridian in V1 is near the fundus of the calcarine sulcus. Individual aura reports show an asymmetric propagation to either the upper or lower visual field quadrant [1], [2], [3], [6]. If the visual field defect falls behind in one visual quadrant, this could indicate that *M* is larger in this quadrant. Indeed, anatomical data suggest that V1 proceeds farther anteriorly in the lingual gyrus [32], [42], which suggests that more cortical surface is devoted to upper quadrant, however, fMRI data show that the dorsal and ventral compartments of V1 are at least similar in absolute extent measured in geodesic distance [40].

#### Ending

Visual aura symptoms stop in the periphery of the visual hemifield. The extreme periphery of the visual hemifield is represented at the anterior boundary of V1 close to the T-shaped or sometimes Y-shaped junction of the calcarine sulcus and the medial part parieto-occipital sulcus. Such a junction might act as a diode being transparent for wave propagation only in one direction, but not in the other. Critical properties of excitation waves on curved surfaces that lead to a curvature-dependent loss of excitability have been studied in BZ system [30].

The calcarine sulcus as a major sulcus is composed of smaller gyral folds and sulci indents resulting in a complex surface. While the gross morphology of the calcarine sulcus can determine the basic course of particle-like wave propagation, it is this individually complex surface that needs to be considered if precisely recorded perimetric data of visual aura progression are compared with anatomy. Furthermore, only rather sharp deformations of the cortical surface can directly induce a critical deformation in the wave front above which propagation is then blocked [31]. The effect of smaller gyral folds and sulci is considered in the next subsection.

### fMRI retinotopy and perimetric aura data

**Figure 3:**
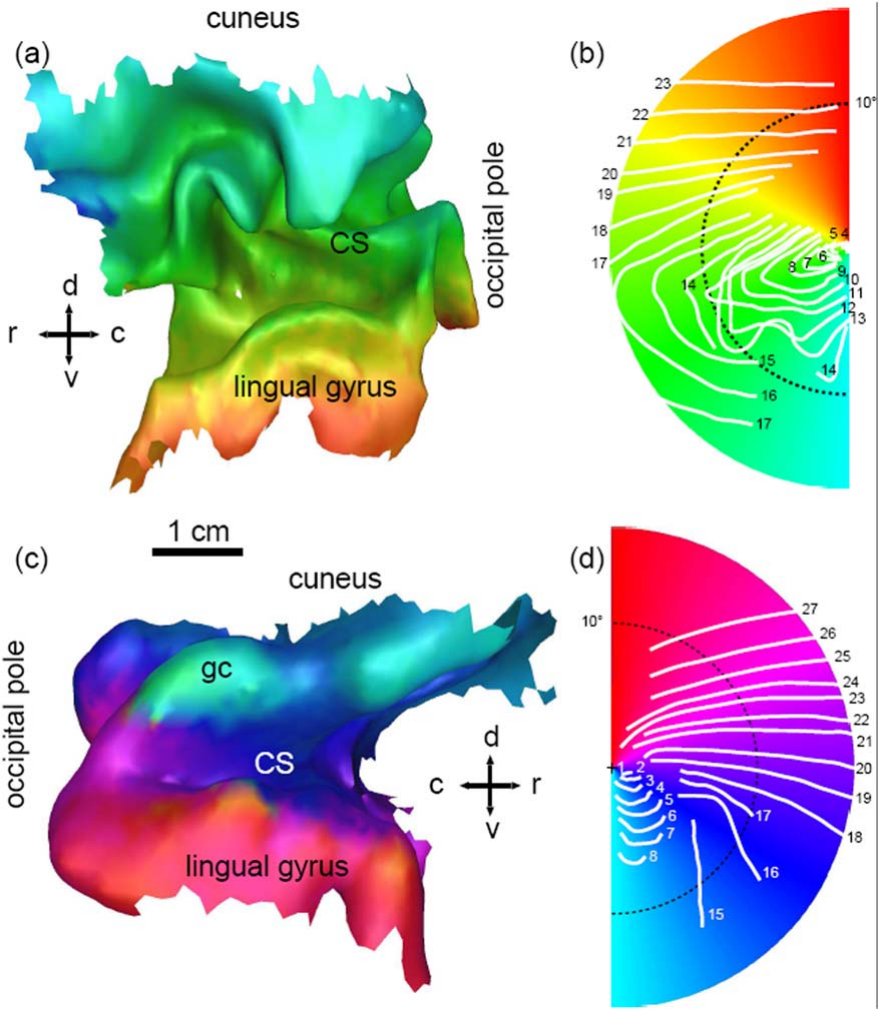
3D form of primary visual cortex (V1). The representation of the azimuthal coordinate of the two visual hemifields is given by the hue, value, saturation color model: (a) right V1 (b) left visual hemifield (c) left V1 (d) right visual hemifield. The current position of the visual field defect, occurring during two different migraine aura attacks and each exclusively in one visual hemifield, are indicated by white lines, with numbers denoting the time in minutes after onset.

To investigate the effects of small gyrification pattern on reaction-diffusion waves, the 3D form of V1 and its retinotopic map was obtained by fMRI from a migraineur (PVV) who has made precise perimetric recordings of his visual aura [43]. In Fig. 3 (a), the right V1 is shown. The color of the right V1 codes the azimuthal angle of the contralateral left visual hemifield by a half color wheel (see Fig. 3 (b)) of the hue, value, saturation color model, i. e., in counterclockwise direction from red (upper hemimeridian) via light green (horizontal hemimeridian) to cyan (lower hemimeridian). The rostral/caudal (r, c) and dorsal/ventral (d, v) directions are indicated by crossed arrows.

The dorsal bank of the right calcarine sulcus is noticeable heavily ramified with small gyral folds and sulci indents. The progression pattern of the visual field defect in the lower visual quadrant shows accordingly a rather complex pattern. The spatial progression is marked in Fig. 3 (b) by drawing with white lines the current position of the propagating field defect, i. e., the leading edge of the scintillating zigzag pattern, at one minute intervals within 24 minutes. The wave runs from minute 4 to 13 in the lower visual quadrant and this quadrant is mapped, as can be seen by the color code, onto the dorsal bank of the calcarine sulcus. Partly the wave pulsates back and forth between 11–13 minute and eventually terminates in the lower end of this visual quadrant in an excitation block, but continues to propagate within the upper quadrant.

In Fig. 3 (c), the left V1 is shown with the color coding azimuthal angles of the contralateral right visual hemifield given by the other half of the hue, value, saturation color model, i. e., in counterclockwise direction from cyan (lower hemimeridian) via dark magenta (horizontal hemimeridian) to red (upper hemimeridian). Marked with white lines at one minute intervals, the spatial progression of the visual aura in the right visual hemifield is shown in Fig. 3 (d). As can be seen by the color code, the wave runs from minute 1 to 8 over a gyral crown (gc) as part of the cuneus. Between minute 8 to 15 the wave disappeared, but reappeared at minute 15 propagating upwards in the visual field for a duration lasting 12 minutes being approximately parallel to visual hemimeridians, i. e., running from the dorsal to the ventral bank of the calcarine sulcus and ending on the anterior edge of the lingual gyrus.

## Discussion

The crescent shaped aura pattern, as shown in Fig. 1 (a), is often reported [1], [2], [3], [4], [5], [6] but the phenomenology of migraine aura is much richer as documented by the variety of illustrations and descriptions collected on the Migraine Aura Foundation website (www.migraine-aura.org). In a single migraine aura attack, migraineurs can also experience diverse visual, as well as sensory, motor and language disturbances [44], [45]. This variety clearly indicates that other areas beside early visual cortex can be affected, even cortical areas outside the occipital lobes, and it therefore seemingly supports the idea that the process causing the aura can engulf all of posterior cortex on its course, like a cortical SD wave observed in animal experiments.

Schematic drawings similar to Fig. 2 (a) illustrate engulfing spatio-temporal wave patterns. Such illustrations are found in modern textbooks of headache [46] and appeared first in Lauritzen's seminal paper spearheading the SD theory of migraine aura [47]. They became paradigmatic for migraine with aura. However, they might need to be revised, as we show. First it must be noted that the hypothesis of such a spatio-temporal development of a migraine attack was likely motivated by the SD pattern on the cortex of animals with cortices much smaller than the human cortex . If the SD wave occupies similar areas in different species, then the SD wave that covers most of the hemisphere in small animals would occupy only a small part of the human V1 area. This probably is the case, because the propagation velocity of SD is similar in all cortices. As a consequence, the SD pattern cannot simply be scaled with the cortex size.

The activity pattern causing crescent shaped aura is remarkably similar to a particle-like wave segment on the cortical surface, a pattern that exists only in cortex being weakly susceptible to SD. For example, the open end of the wave segment shown in Fig. 1 (b) slightly grow within the observed 10 minutes interval without curling in to form a reentrant pattern. This indicates a susceptibility value very close to one. In contrast, reentrant SD pattern observed in retina rotate with a period of 2.5 minutes [20] which would correspond to the passage of four waves within 10 minutes.

Other factors also support the concept that human cortex is only weakly susceptible to SD, maybe foremost that susceptibility becomes the lower the higher up the species is in the phylogenetic tree. Another clear indication is that SD propagation is modulated by cortical morphology, as can be seen in Fig. 3. Similar pattern were also observed for the gyrencephalic feline brain [48], but there the primary SD wave engulfed the hemisphere and only succeeding secondary waves remained within the originating gyrus and were more fragmented. Since secondary waves run into partly refractory tissue, susceptibility to SD is decreased.

The engulfing wave pattern was originally attributed to the smooth architecture of the cortex of rats and rabbits. It has been debated whether SD can occur in the highly convoluted cortex of humans, until spatial and temporal events were followed using high-field functional MRI [49] demonstrating that at least eight characteristics of SD are present and the events are time-locked to percept onset of the aura in human cortex. However, the precise spatio-temporal course of the events is still ambiguous. Much of posterior cortex, including several retinotopically organized visual areas, showed simultaneous activation during much of the period of the aura, while the percept in the visual hemifields is reported to be more spatially confined.

As already noted by Wilkinson [50] this mismatch in fMRI data and aura percept can be explained by at least two alternatives: (i) either SD engulfs all of posterior cortex. Then only a subset of this activation results in sensory awareness. Or (ii), the spread of the SD wave is, in contrast to the fMRI data, more limited in extent. Then the rest of the observed activation in adjacent cortical areas represents synaptic activation through feed-forward and feedback circuitry. While (i) is in agreement with observed cortical SD wave patterns in animals, it opens up questions about the nature of the often reported limitation to spatially confined crescent-shaped visual field defects. In (ii) spatially confined SD waves causing corresponding field defects are simply postulated [50].

If SD in human is more limited in extent, the mismatch with animal data needs to be addressed. To reconcile this, we provide a theoretical framework, which is, moreover, of practical use to both experimental neuroscientists and clinicians. We propose a susceptibility scale σ based on nonlinear bifurcation analysis. Not unlike the Celsius temperature scale, the term *susceptibility to SD* is made a precise scale by a two-point definition, i. e., two macroscopically observable cortical states at which a phase transition in SD pattern formation occurs. Before we describe the relevance and applicability of this scale in the following, we end this paragraph briefly discussing other possible definitions of tissue excitability. Using detection or discrimination measures or stimuli reported to trigger migraine (striped patterns or flickering lights) one finds differences between people with and without migraine, which is attributed to abnormal cortical processing in migraine, described by hyper- or hypoexcitability, heightened responsiveness, a lack of habituation and/or a lack of intra-cortical inhibition [50], [51]. Such statements on cortical excitability in migraine are based on psychophysical measurements of visual function, in particular early aspects of visual processing. It remains to be investigated how abnormal cortical processing changes susceptibility to SD. Although it seems tempting to suggest that cortical hyperexcitability increases susceptibility to SD or even that neurons prone to hyperexcitabilty trigger SD, such a simple relation cannot be expected.

The weakly susceptible state (1>σ>0) of human cortex to SD can be achieved in experimental migraine models if the tissue is treated reducing excitability towards the gray marked regime in Fig. 2. The procedure to find this regime experimentally is described in the Methods section for retinal SD. Retinal SD is accompanied with an intrinsic optical signal that makes precise spatio-temporal recordings of the evolutionary SD pattern possible. Similar precise spatio-temporal recordings have been made in cortex using a fluorescent, voltage-sensitive dye [52].

We predict that effects of antimigraine drugs depend on the susceptibility range they are tested in, because the dynamical behavior of a nonlinear system changes drastically when crossing a bifurcation point. Antimigraine drugs tests and tests to unravel the mechanism of SD in retina [53], [54], [55] have been performed far away from the regime (1>σ>0). This can be shown by precisely measuring in this system the complex meandering pattern of spiral SD [20]. On the σ scale, obtained from the generic FitzHugh-Nagumo model, these pattern occur above σ>2 and are separated by two further bifurcations [22]. In general, SD experiments are performed in the most prone tissue regions where SD can more easily be observed. This might remind of Watzlawick's man searching for his keys under the streetlight rather than where he lost them [56].

Furthermore, our results supports the idea that SD could activate the trigeminovascular system that generates and maintains migraine pain [57] even in diagnosed forms of *migraine without aura*. For susceptibility values below the weakly susceptible regime, the model predicts spatio-temporal SD pattern that do not break away from an initially restricted focus. We can draw a direct analogy to clinically silent epilepsy caused by interictal activity that does not break away from a focus. Likewise, previously proposed silent aura, in which “some migraineurs exhibit blood flow ‘fingerprints’ of CSD [cortical SD] and aura but are subjectively unaware that the phenomenon is propagating” [15], may be explained by localized SD patterns occurring at the one end of the increased dynamical repertoire that emerges if being close to a bifurcation.

## Methods

### Susceptibility scale in experimental and mathematical models

**Figure 4:**
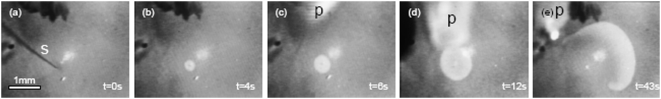
Creation of an SD wave segment with free open ends in submerged chicken retina. (a) Mechanical stimulation with sharp glass needle s, (b) circular SD wave evolves, (c)–(d) local application of Mg^2+^ via pipette p, (e) wave propagation is locally blocked and consequently SD front brakes open and curls in to form a spiral at the lower open end, while the upper open end is guided by the Mg2+-pipette to the border of the retina where it attaches.

A two-point definition is used for calibrating the newly introduced susceptibility scale σ. These two points are macroscopically observable states. We shortly describe an experimental procedure to measure such states. A precise determination of these two states in an animal model of SD is, however, beyond the scope of our proof of concept. The propagation boundary ∂*R* (σ = 1) can be obtained by changing the tissue excitability until open wave segments stop curling in to form reentrant SD waves with freely rotating open ends forming two centers (spiral SD) [58]. For instance, to obtain an SD wave segment in submerged chicken retina (for details, see Ref. [20]), an initially closed circular SD wave front can be broken (at a diameter of about 0.75 mm) by local application of 0.5 ml Ringer solution through a pipette (tip diameter 0.5 mm) containing a tenfold raised Mg2+ concentration (10 mM) (Fig. 4). Retinal SD is described in detail in Ref. [59].

**Figure 5:**
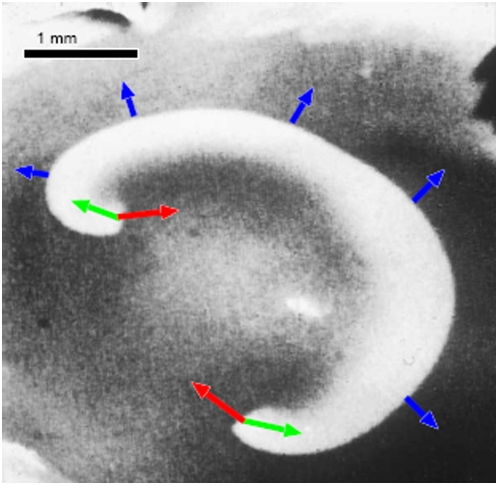
Retinal SD wave segment propagating (blue arrows) with free open ends that grow (red arrow) and therefore curl in to form a double spiral. At lower susceptibility values, reaction-diffusion models of SD predict that open ends retract (green arrows) and the wave vanishes.

In Fig. 5 a retinal SD wave segment is shown that evolves into a double spiral (σ>1). The mathematical model (see below) predicts that after crossing at σ = 1 the propagation boundary ∂*R*, open ends of the wave segments retract (direction indicated by green arrows in Fig. 5) and the SD wave eventually vanishes. The Mg2+ concentration in Ringer at which this transition occurs is in this experimental set-up difficult to determined, because the initial raise in Mg2+ needed to break the circular wave front cannot sufficiently fast be washed out. However, it is known that lowering calcium concentration to 0.5 mM and increasing magnesium to 2.0 mM turns the tissue absolute refractory to SD [60], which corresponds to the regime σ<0 and giving a lower bound of σ = 0.

The locus of ∂*R* as a function of excitability represents a critical perturbation threshold [18], separating an attractor characterized by spiral waves from an attractor characterized by the uniform physiological steady state of the cortex. Such a threshold, called rotor boundary ∂*R* must exist if spiral SD waves occur in the tissue and therefore the existence of the rotor boundary ∂*R* is independent of the particular model that describes the pattern formation process. The locus of the propagation boundary ∂*P* can be obtained similarly by decreasing further the tissue excitability until reentrant SD waves collapse even if their open ends are attached to either the border of the retina or a lesion (circling SD [61]).

In mathematical models of SD, the critical points ∂*R* and ∂*P* are found by bifurcation analysis. Some SD models investigate the local ignition of SD by mathematical models of single cells and their surrounding compartments [62], [63]. Those models lack a spatial extension beyond the cell size. They cannot yet address the clinically relevant question whether a local ignition stays confined or breaks away but such microscopic models help to understand the pathophysiological mechanism of SD and if they will be extended by a spatial coupling, such as a diffusion term, also those models become accessible to the bifurcation analysis described in the following.

We exemplify with a standard reaction-diffusion scheme of activator-inhibitor type the determination of the location of ∂*R* and ∂*P* in the parameter space of this model and how to obtain from a parameter value the susceptibility scale σ. By choosing an activator-inhibitor type SD model, we assume that all quantities with a positive feedback loop can be lumped together, such as extracellular potassium concentration and inward currents [64], [65]. They become a single *activator* variable *u*. The rate of change in *u* is given by a single nonlinear reaction rate *f*. Likewise, a single inhibitor variable *v* represents the recovery processes with reaction rate *g*. Processes represented by inhibitor kinetics are, amongst others, the effective regulation of by the neuron's - ion pump and the glia-endothelial system. The general form of a reaction-diffusion equation is then
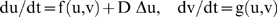
(1)where the term DΔu represents the spatial coupling of the local dynamics by diffusion of *u* with diffusion coefficient *D*.

The variety of macroscopic reaction-diffusion pattern in *u* and *v*, such as spirals and retracting waves, is largely independent of the specific reaction rates *f*(*u*,*v*) and *g*(*u*,*v*), as long as the local dynamics (*D* = 0) show all-or-none type behavior. To obtain the scale σ shown in Fig. 2 we chose the FitzHugh-Nagumo equations and *g*(*u*,*v*) = (*u*+β)/25, where β is a threshold parameter that selects the pattern. These equations can also be derived as an extended Grafstein-Hodgkin model of SD [66].

In the extended Grafstein-Hodgkin model of SD, particle-like waves can be stabilized with a control term that changes β as a linear function of wave size, a feedback mechanism first proposed for chemical BZ waves [18]. At the limit of large particle-like waves is reached (critical fingers [23], [24]). The propagation boundary is found by transforming Eqs. 1 into a co-moving frame and determine the largest value for which bounded profile solutions exist [26]. The susceptibility scale, as a linear function of β with the defining points σ = 1 and σ = 0 corresponding to ∂*R* and ∂*P*, respectively, is then obtained by
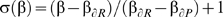
This formula is independent on the specific choice of β as a parameter to change excitability, e. g., in an experimental system β_∂*R*_ and β_∂*P*_ could be taken as the concentration of Mg^2+^ as described above. If more then one parameters are accessible to change tissue excitability a shortest, i.e., metrical, distance between ∂*R* and ∂*P* can be defined via pharmacokinetic-pharmacodynamic models [26].

### Perimetric recordings and retinotopic mapping

The perimetric data shown in Fig. 1 were taken from Lashley's precise drawings published in 1941 [1]. The radial coordinate (eccentricity) of the crescent shaped aura pattern in the right visual hemifield was calibrated assuming the blind spot (not shown in Fig. 1) at 10 degrees eccentricity. The eccentricity is then obtained assuming the percept is projected to a flat tangent plane with respect to the center of the spherical visual field. This tangent plane serves as the canvas to draw the aura percept. The azimuthal coordinate can be taken directly form the drawing in the tangent plane.

The flat retinotopic map in Fig. 1 (b) was created by using the monopole map, that is, the complex logarithm w = A log(z/E_2_+1) with the cortical magnification parameter E_2_ = 0.75 and *A* = 17.3 adjusted to human data [67]. The complex coordinates *z* and *w* describe locations in the visual field and in the cortical domain, respectively. The magnitude of *z* is the visual eccentricity θ and its argument φ is the azimuth. The real and complex parts of *w* are Cartesian coordinates on the cortical surface. From the monopole map it follows that the linear cortical magnification factor along the horizontal hemimeridian is M(θ) = A/(θ+E_2_).

The perimetric data shown in Fig. 3 were provided by a participant (PVV) who fulfills the International Headache Society criteria for the diagnosis of migraine with aura. As a research engineer he trained himself to make precise recordings during his migraine with aura attacks and documented over 350 aura episodes over 10 years [43]. To compare the topography of the visual aura with anatomical landmarks of the cortex, the retinotopic organization in the visual cortex was obtained with functional magnetic resonance imaging (fMRI). The data were acquired in a 3-Tesla scanner, using echoplanar imaging as described in Refs. [49], [68].
